# Small secreted peptides encoded on the wheat (*Triticum aestivum* L.) genome and their potential roles in stress responses

**DOI:** 10.3389/fpls.2022.1000297

**Published:** 2022-09-21

**Authors:** Dongdong Tian, Qi Xie, Zhichao Deng, Jin Xue, Wei Li, Zenglin Zhang, Yifei Dai, Bo Zheng, Tiegang Lu, Ive De Smet, Yongfeng Guo

**Affiliations:** ^1^ Tobacco Research Institute, Chinese Academy of Agricultural Sciences, Qingdao, China; ^2^ Key Laboratory of Horticultural Plant Biology of Ministry of Education, College of Horticulture and Forestry Science, Huazhong Agricultural University, Wuhan, China; ^3^ Biotechnology Research Institute, Chinese Academy of Agricultural Sciences, Beijing, China; ^4^ Department of Plant Biotechnology and Bioinformatics, Ghent University, Ghent, Belgium; ^5^ VIB Center for Plant Systems Biology, Ghent, Belgium

**Keywords:** small secreted peptides, wheat, stress response, drought stress, salt stress, heat stress

## Abstract

Small secreted peptides (SSPs) are important signals for cell-to-cell communication in plant, involved in a variety of growth and developmental processes, as well as responses to stresses. While a large number of SSPs have been identified and characterized in various plant species, little is known about SSPs in wheat, one of the most important cereal crops. In this study, 4,981 putative SSPs were identified on the wheat genome, among which 1,790 TaSSPs were grouped into 38 known SSP families. The result also suggested that a large number of the putaitive wheat SSPs, Cys-rich peptides in particular, remained to be characterized. Several *TaSSP* genes were found to encode multiple SSP domains, including CLE, HEVEIN and HAIRPININ domains, and two potentially novel TaSSP family DYY and CRP8CI were identified manually among unpredicted TaSSPs. Analysis on the transcriptomic data showed that a great proportion of TaSSPs were expressed in response to abiotic stresses. Exogenous application of the TaCEPID peptide encoded by TraesCS1D02G130700 enhanced the tolerance of wheat plants to drought and salinity, suggesting porential roles of SSPs in regulating stress responses in wheat.

## Introduction

As multicellular organisms, cell-to-cell communication in plants is critical in coordinating growth and development, as well as in tolerating and surviving uncomfortable environmental conditions (Vie et al., 2015). Plant small secreted peptides (SSPs) are signaling molecules derived from larger inactive precursor proteins, which are generally less than 250 amino acids (aa) in length, and can be actively secreted out of cells as crucial intercellular communication messengers (Lease and Walker, 2006; Matsubayashi and Sakagami, 2006; Olsson et al., 2019). SSPs are demonstrated as key regulators in a diverse array of plant growth and development processes, including root growth, meristem maintenance, vascular development, casparian strip formation, stomatal development, floral organ abscission, pollen tubes growth, stress acclimation, defense response, senescence etc. (Motose et al., 2009; Katsir et al., 2011; Aalen et al., 2013; Czyzewicz et al., 2013; Mosher et al., 2013; Marmiroli and Maestri, 2014; Murphy and De Smet, 2014; Sauter, 2015; Nakayama et al., 2017; Zhang et al., 2022).

SSP precursors share a number of common features: an N-terminal signal peptide important for secretion, a shorter C-terminal conserved domain (active mature peptide), and a variable part in the middle (Matsubayashi, 2011). The SSP precursor itself can be functional, such as Cysteine-rich secretory protein, antigen 5 and PR-1 (CAP)-derived Peptide 1 (CAPE1). Unfunctional SSP precursors need to be proteolytically processed to release mature SSPs (Tavormina et al., 2015; Hu et al., 2021). SSPs can be divided into three categories based on their mature forms: the first category is post-translationally modified (PTM) peptides, which consists of less than 20 aa in their mature forms and specific amino acids are modified, such as C-TERMINALLY ENCODED PEPTIDE (CEP) (Ohyama et al., 2008), CASPARIAN STRIP INTEGRITY FACTOR (CIF) (Doblas et al., 2017; Nakayama et al., 2017), CLAVATA3/EMBRYO SURROUNDING REGION (CLV3/CLE) (Fletcher et al., 1999; Cock and McCormick, 2001; Gao and Guo, 2012), INFLORESCENCE DEFICIENT IN ABSCISSION (IDA) (Butenko et al., 2003), ROOT MERISTEM GROWTH FACTOR (RGF) (Matsuzaki et al., 2010), PHYTOSULFOKINE (PSK) (Matsubayashi and Sakagami, 1996) and PLANT PEPTIDE CONTAINING SULFATED TYROSINE1 (PSY1) (Amano et al., 2007) families. The second category is Cys-rich peptides (CRP), which contain an even number (2-16) of Cys residues in their mature forms. Disulfide bridges formed in pairs of conserved cysteines help CRPs to fold into their final active conformations (Broekaert et al., 1997). Examples of CRPs include RAPID ALKALINIZATION FACTOR (RALF) (Pearce et al., 2001), STERILITY LOCUS CYSTEINE-RICH PROTEIN (SCR/SP11) (Schopfer et al., 1999; Takayama et al., 2000) and EPIDERMAL PATTERNING FACTORS (EPFs) (Hara et al., 2007) families. The third category contains non-Cys-rich and non-PTM peptides, such as systemin (SYS), and PLANT ELICITOR PEPTIDES (PEP) families (Tavormina et al., 2015; Hu et al., 2021).

During the past decade, an large number of SSPs have been identified in various plant species. 33809 ORFs encoding SSPs with length from 25 to 250 aa were predicted in the Arabidopsis genome (Lease and Walker, 2006). Yang et al. obtained 12852 ORFs encoding proteins of 10-200 aa in length from *P. deltoids* (Yang et al., 2011). 101048 putative ORFs for SSPs with length between 25 and 250 aa were identified in rice (Pan et al., 2013). 1491 putative SSPs that were less than 200 aa in length were identified from the maize genome (Li et al., 2014). 236 SSPs involved in plant immunity with length less than 250 aa were identified in rice (Wang et al., 2020). 4439 SSP-encoding genes with protein length less than 250 aa were identified in *M. truncatula* (Boschiero et al., 2020). However, little is known for SSPs in common wheat (*Triticum aestivum* L., 2*n* = 6*x* = 42; AABBDD genome), except one previous report, which describes the identification of small secreted proteins in wheat and demonstrated potential roles of small secreted proteins in responding to *Septoria tritici* bloth (SBT). No comprehensive identification and classification of peptide domains was reported however (Zhou et al., 2020).

Unlike animals, plants are sessile organisms inescapably exposed to abiotic stress in their living environment (Wang et al., 2020). Therefore, plants have developed complex mechanisms to sense and respond to unfavorable growth environments. As one of the most important cereal crops, wheat is constantly exposed to abiotic stresses such as drought, salinity, heat and cold, which negatively affect plant development and agricultural production. Thus, it is essential to understand how wheat adapts and survives in stressful environments. SSPs have recently emerged as key signaling molecules in stress responses (Chen et al., 2020c). In Arabidopsis, RALFL8 is induced by drought stress, leading to cell wall remodeling (Atkinson et al., 2013); CAPE1 negatively regulates plants’ response to high salinity stress (Chien et al., 2015); CLE25 is induced in roots under dehydration, triggering ABA biosynthesis in leaves to prevent water loss by regulating stomatal closure (Takahashi et al., 2018); Pep3 is involved in salinity stress tolerance (Nakaminami et al., 2018); CEP5-mediated signaling is relevant for osmotic and drought stress tolerance (Smith et al., 2020); the PIP3-RLK7 signalling module regulates plant salt tolerance by activating the MPK3/MPK6 cascade in Arabidopsis (Zhou et al., 2022). More recently, secreted peptides SMALL PHYTOCYTOKINES REGULATING DEFENSE AND WATER LOSS (SCREWs) were shown to be involved in regulating stomatal closure in response to ABA and microbe-associated molecular pattern (MAMP) signals in Arabidopsis (Liu et al., 2022).

Most molecular functional studies related to the role of SSPs in stress tolerance have been carried out in model plants such as Arabidopsis, rice and tomato, while limited studies were conducted in wheat. In this study, wheat SSPs (TaSSPs) were identified and expression changes of *TaSSP* genes upon drought, heat and salt stresses were analyzed to predict their potential roles in stress responses. We also studied the roles of a TaCEP peptide in regulating drought and salt responses through exogenous peptide application. The results provide new information of wheat SSPs and their potential functions in response to abiotic stresses.

## Materials and methods

### Identification of SSP-encoding genes in wheat

All of the *Triticum aestivum* protein sequences were downloaded from EnsemblPlants (http://plants.ensembl.org/index.html). Subsequently, a multi-step procedure was performed to identify wheat SSPs based on the common structure and sequence features of SSP precursors as described in **Figure S1A**: full length protein sequences of TaSSPs were used to predict signal peptide using the SignalP-5.0 software (https://services.healthtech.dtu.dk/service.php?SignalP) (Almagro Armenteros et al., 2019), and protein sequences with signal peptide removed were used to predict transmembrane (TM) domain using the TMHMM v2.0 software (https://services.healthtech.dtu.dk/service.php?TMHMM-2.0) (Krogh et al., 2001). Endoplasmic reticulum (ER) docking proteins were identified based on the C-terminal conserved domain K/HDEL (Lys/His-Asp-Glu-Leu) (Napier et al., 1992). For TaSSP-encoding genes with multiple transcripts, the longest transcript was used for further analysis.

### TaSSP prediction and classification

The putative TaSSP-encoding genes were predicted and classified into known or likely-known SSP families based on the *Medicago truncatula* Small Secreted Peptide Database (MtSSPdb; http://mtsspdb.noble.org/database/) (Boschiero et al., 2020). The predicted SSPs were further screened based on their homology with SSP HMM profiles and homology with protein sequences of known SSPs. Gene IDs of the resulted SSPs were used to perform blast in *Triticeae*-*GeneTribe* (http://wheat.cau.edu.cn/TGT/m7/?navbar=Homologues), with the assembly of *Triticum aestivum* (IWGSC RefSeqv1.1) (Chen et al., 2020b). Gene description of TaSSPs was obtained using gene ID by GeneDescription tool on *Triticeae*-*GeneTribe* (http://wheat.cau.edu.cn/TGT/m19/?navbar=ByGeneID). The number of cysteine residues in each TaSSP without signal peptide was recorded for prediction of CRP type SSPs.

### Bioinformation analysis

Chromosomal localization of *TaSSP* genes was visualized using the TBtools software (Chen et al., 2020a). Phylogenetic trees containing the full-length amino acid sequences for PIP and CEP precursor proteins from both Arabidopsis and *T*. *aestivum*, for DYY, CRP8CI precursor proteins from *T. aestivum* were constructed using the MEGA 5.05 software (Hall, 2013) respectively, with the following parameters: alignment, ClustalW, phylogeny construct or test, Maximum Likelihood Tree, number of bootstrap replications = 1000. Subsequently, the phylogenetic tree was modified with the iTOL online tool (https://itol.embl.de/itol.cgi) (Ciccarelli et al., 2006). Multiple sequence alignment was performed using the DNAMAN v6.0 software (Lynnon Biosoft, Quebec, Canada). Weblog-Create Sequence Logos (http://weblogo.berkeley.edu/logo.cgi) were used for comparative analysis of domain conservation and for conservation analysis of each amino acid site of PIP/CEP domains (Crooks et al., 2004). GO analysis was performed with agriGO v2.0 (http://systemsbiology.cau.edu.cn/agriGOv2/index.php) (Tian et al., 2017).

### *In silico* gene expression analysis

The RNA-Seq-derived data of *TaSSP* genes were downloaded from WheatOmics 1.0 (http://wheatomics.sdau.edu.cn/) (Ma et al., 2021). Expression data for drought, heat (Liu et al., 2015) and salt stress responses (Zhang et al., 2016) were obtained from IWGSC Annotation v1.1. The TPM values ≥ 1 were considered meaningful. A TPM ratio of stress treatments vs. corresponding control of ≥ 2 was considered significantly upregulated. *TaSSPs* with a TPM value < 1 in control and ≥ 1 in a particular stress treatment were considered specifically upregulated genes. For salt stress, two groups of RNA-seq data from Chinese Spring and Qing Mai 6 were obtained and similar gene expression patterns were observed between the two varieties. The results from Chinese Spring was used for further analysis in this study.

### Plant materials and growth conditions

Wheat cultivar Ji Mai (JM) was used in this study. Wheat seeds were surface-sterilized in 1% sodium hypochlorite (NaClO) for 15 min, followed by washing in sterile distilled water for 3 times to fully remove NaClO, and then put on wet filter paper in sterile Petri dishes for 2 d at 20°C. Uniformly germinated seeds were tansferred into 96 hole hydroponic boxes (310 mm × 290 mm × 180 mm) with Hoagland solution to be cultured in a growth chamber with 22°C/18°C (day/night), 16 h/8 h (day/night) and 50% humidity. After 7 days, the plants were subjected to heat stress (40°C), drought stress (20% (m/v) PEG-6000), or combined heat and drought stress treatments for 1 h and 6 h, respectively. Salt stress (150 mM NaCl) was applied for 6 h, 12 h, 24 h and 48 h, respectively. Plants grown under normal growth conditions were used as controls. All experiments were performed with three biological replicates. Leaves were collected separately at 1 h and 6 h for heat and drought stresses, roots were collected separately at 6 h, 12 h, 24 h and 48 h for salt stress, all samples were frozen immediately in liquid nitrogen and stored at −80°C for further use (Liu et al., 2015; Zhang et al., 2016).

### qRT-PCR

Total RNA from leaves and roots was extracted using TRIzol reagents (CWBIO, Taizhou, China) according to the manufacturer’s instructions and RNA concentration was measured using a NanoDrop^®^ 2000 spectrophotometer (Thermo Fisher Scientific, Wilmington, Delaware, USA). The RNA samples with A_260_/A_280_ ratios ranged from 1.8 to 2.1 and A_260_/A_230_ ratios ≥ 2.0 were stored at −80°C for future use. 1 μɡ of total RNA was used as templates to synthesize cDNA using the HiScript II One Step RT-PCR Kit (Vazyme, Nanjing, China). qRT-PCR reactions were then performed using the SYBR^®^ Green Premix Pro Taq HS qPCR Kit (Rox Plus) (Accurate Biology, Changsha, China). A standard 2-step amplification protocol was run in a QuantStudio 3 Real-Time PCR System (Thermo Fisher Scientific, Wilmington, Delaware, USA). The comparative C_T_ method (△△Ct method) was used for relative quantification of real-time PCR results (Pfaffl, 2001). *ACT2* (TC234027), *CYP18-2* (AY456122.1), *TaFNR1I* (AJ457980.1), *ACT* (AB181991) and *UBI* (AY297059) were selected as candidate reference genes (Tenea et al., 2011; Dudziak et al., 2020), and *TaFNR1I* was used eventually. All gene specific primers (**Table S1**) were designed using the Primer Premier 5.0 software (www.PremierBiosoft.com). All PCR reactions were run in three biological replicates and two technical replicates.

### Peptide synthesis and treatments

The TaCEP1D peptide derived from the TraesCS1D02G130700-encoded proprotein (**Table S1**) was chemically synthesized by GenScript Biotech Corporation (Nanjing, China), with a purity ≥ 90% (w/w). Peptides were dissolved in double distilled water (ddH_2_O) at a stock concentration of 1 mɡ/mL and stored at −80°C for future use. Uniformly germinated seeds were transferred into 10 cm-diameter pots (16 plants in each pot) or 32 well trays (four plants in each well) with equal amounts of matrix soil respectively, and grown in a growth room at 22°C/18°C (day/night), 16 h/8 h (day/night) with a humidity of 50%. The plants were watered every other day with 80 mL distilled water for individual pot and 350 mL for each tray. Ten days after transplanting, drought stress was performed through water wthdrawal, salt stress was performed by adding 300 mM NaCl solution with the frequency of 80 mL every other day, and control plants were grown with normal water supply. For peptide treatments, distilled water or TaCEP1D peptide was sprayed to wheat leaves twice every day. Each treatment consisted of three biological replicates and each replication contained 16 plants.

For detached leaf treatments, the first leaves from two-week-old wheat plants were cut into 2-3 cm segments, placed on wet filter paper in Petri dishes (6 cm) that contained 2 mL distilled water for control, 200 mM NaCl for salt stress, and 20% (m/v) PEG-6000 for drought stress. The TaCEP1D peptide was added to the Petri dishes with the final concentrations of 0.5 μM and 1μM, respectively. The Petri dishes were sealed and incubated in a growth chamber at 22°C/18°C (day/night), 16 h/8 h (day/night) with 50% humidity. Each treatment was performed with three biological replicates, and each replication contained eight leaf segments.

### Morphological analysis and physiological measurements

The wheat plants and detached leaves were photographed with a Canon EOS 7D to obtain high-resolution images. Shoot length and fresh weight were measured. Total chlorophyll contents and electrolyte (ion) leakage rates were determined as previously reported (Zhang and Guo, 2018). Fv/Fm was detected using a Modulated Chlorophyll Fluorometer (OPTI-SCIENCES OS1p, USA). The O^2-^ detection, SOD activity detection and MDA content measurement were performed using 0.1% NBT solution (1 mɡ/mL, pH 7.8, Coolaber, Beijing, China), Superoxide Dismutase Activity Detection Kit (Solarbio, Beijing, China) and Micro Malondialdehyde (MDA) Assay Kit (Solarbio, Beijing, China), respectively, each with three biological replicates.

### Statistical analysis

All the experiments in this study were performed with three biological replicates. Significance of difference was determined *via* the t-test analysis with double sample variance assumption (*p* < 0.05).

## Results

### Identification of SSPs in *T*. *aestivum*

To identify SSPs in wheat, we downloaded the protein sequences of *T. aestivum* from the EnsemblPlants platform (http://plants.ensembl.org/index.html). Among the 38,899 small proteins with no more than 250 amino acid residues, 5,493 proteins were predicted by SignalP 5.0 (https://services.healthtech.dtu.dk/service.php?SignalP) to have a N-terminal signal peptide for the secretory pathway (Almagro Armenteros et al., 2019). The number of proteins was reduced to 5,111 after removing the proteins containing a transmembrane domain predicted by TMHMM v2.0 (https://services.healthtech.dtu.dk/service.php?TMHMM-2.0) (Krogh et al., 2001) and the potential endoplasmic reticulum docking proteins based on the presence of KDEL or HDEL domains at C-termini (Napier et al., 1992). Finally, 4,981 potential TaSSP-encoding genes were identified after deleting the truncated proteins (**Figure S1A**, **Table S2**). Length of most of the putative TaSSP proproteins ranges from 61 to 230 residues, with only ten SSPs being shorter than or equal to 60 residues (**Figure S1B**). The molecular weight and *p*I of the TaSSPs range from 4.66 KDa to 28.8 KDa and 3.19 to 13.07, respectively (**Table S2**). Chromosomal localization analysis showed that the 4,981 putative *TaSSP* genes are distributed throughout all chromosomes on the wheat genome, with a large proportion located at the ends of the chromosomes (**Figure S1C**). As a heterologous hexaploidy plant with three subgenomes A, B and D, most wheat genes have highly homologous copies on different subgenomes. As a result, a similar number of *TaSSP* genes were identified on subgenomes of each chromosome (**Figure S1C**). Interestingly, the *TaSSP* genes are evenly distributed on each chromosome, except for Chromosomes 4D, 6A, 6B, 6D and 7D which contains less than 200 *TaSSPs*. Due to the preference of *TaSSP* localization at the ends of each chromosome, smaller chromosomes contains larger proportion of *TaSSP*s: 5.2% of the genes on the largest subgenome 5A encode for SSPs, while 28.7% of the genes on the smallest subgenome 4A are *SSP*s (**Figure S1C**).

### Classification of SSPs in *T*. *aestivum*

**Figure 1:**
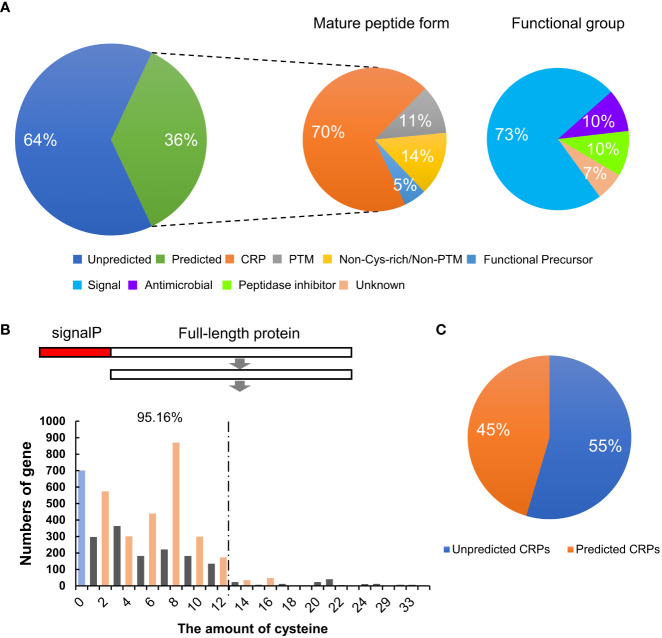
Identification and classification of SSPs in wheat. **(A)** The percentage of different types and functional groups of TaSSPs predicted in MtSSPdb (https://mtsspdb.noble.org/database/). CRP, cysteine-rich peptide; PTM, post-translational modified. **(B)** The amount of TaSSPs with different number of cysteines after deleting signal peptide sequences. SignalP, signal peptide. 95.16% indicates the percentage of TaSSPs with 2, 4, 6, 8, 10 and 12 cysteines to all putative CRPs. **(C)** The percentage of putative unpredicted CRPs and predicted CRPs in MtSSPdb.

Plant SSPs are often grouped into different families based on their conserved peptide domains at the C-termini. To identify members of known plant SSP families in wheat, sequences of the 4,981 potential TaSSPs were used in searching the *Medicago truncatula* Small Secreted Peptide Database (MtSSPdb; http://mtsspdb.noble.org/database/), followed by manual validation. Subsequently, homologue BLAST of the TGT database (Triticeae-GeneTribe, http://wheat.cau.edu.cn/TGT/) was performed to ensure the veracity and entirety of the results of SSP classification. The results showed that 1,790 of the putative TaSSPs (36%) were grouped into 38 known SSP families, including all four peptide forms, CRP (70%), PTM (11%), Non-Cys-rich/Non-PTM (14%) and Functional Precursors (5%) (**Figure 1A**, **Table 1**). Based on predicted functions, peptides of the 38 SSP families have been characterized as signal peptides, antimicrobial peptides, peptidase inhibitors and unknown peptides. Among these, 1335 TaSSPs in 24 families are predicted as signal peptides (**Figure 1A**; **Table 1**).

**Table 1:** Predicted SSP families in wheat.

Class	SSP family name	Description	Mode of action	Number of genes
Post-translationally modified (PTM)	CEP	C-terminally encoded peptide	Signal	13
	CIF	Casparian Strip Integrity Factor	Signal	8
	CLE	Clavata/Embryo Surrounding Region	Signal	88
	GLV/RGF/CLEL	Golven/Root Growth Factor	Signal	9
	IDA	Inflorescence Deficient in Abscission	Signal	18
	PIP	PAMP-induced Secreted Peptide	Signal	18
	PSK	Phytosulfokine	Signal	15
	PSY	Plant Peptide Containing Sulfated Tyrosine	Signal	29
Cysteine- rich	2SA	2S Albumin	Antimicrobial	2
	BBPI	Bowman-Birk Peptidase Inhibitor	Peptidase inhibitor	44
	ECL	Egg Cell 1-Like	Signal	22
	EPFL	Epidermal Patterning Factor-Like	Signal	29
	ES	Embryo Sac (ES)	Signal; Antimicrobial	11
	GASA	Gibberellic Acid Stimulated in Arabidopsis	Signal	36
	HAIRPININ	alfa-HAIRPININ (HAIRPININ)	Antimicrobial; Peptidase inhibitor	4
	HEVEIN	Hevein	Antimicrobial	47
	Kunitz	Kunitz-P trypsin inhibitor	Peptidase inhibitor	14
	LAT52-POE	LAT52/Pollen Ole e 1 Allergen	Signal	84
	LCR	Low-molecular weight Cys-rich	Unknown	16
	MEG	Maternally Expressed Gene	Signal	10
	N26	Nodulin26	Signal	6
	nsLTP	non-specific Lipid Transfer Protein	Signal	463
	PCY	Plantcyanin/Chemocyanin	Signal	240
	PDF	Plant Defensin	Antimicrobial	80
	RALF	Rapid Alkalinization Factor	Signal	38
	RC	Root Cap	Signal	1
	STIG-GRI	Stigma1/GRI	Signal	13
	T2SPI	Potato type II proteinase inhibitor	Peptidase inhibitor	4
	THL	Thionin-like	Antimicrobial	32
	TPD	Tapetum Determinant 1	Signal	47
Non-Cys- rich/Non-PTM	CTLA	Cytotoxic T-lymphocyte antigen-2 alpha	Peptidase inhibitor	19
	GRP	Glycine-rich Protein	Unknown	36
	PhyCys	Phytocystatin	Peptidase inhibitor	93
	PNP	Plant Natriuretic Peptide	Signal	36
	PRP669	Pro-rich Protein Group 669	Unknown	66
	Subln	Subtilisin inhibitor	Peptidase inhibitor	8
Functional Precursor	CAPE	CAP-derived Peptide	Signal	79
	MtSUBPEP	Subtilisin-embedded Plant Elicitor Peptide	Signal	12
	Total			1790

To obtain more information about the TaSSPs that are not grouped in the known SSP families, and since CRP is the most represented type of SSP in wheat, the number of cysteine residues in all of the 4,981 putative TaSSPs proteins without signalP was analyzed (**Table S2**). Putative TaSSPs containing even number (2-16) of cysteine residues at the C-terminal ends (a typical feature of CRPs) were considered putative CRPs. The results suggested that more than 50% of the putative TaSSPs are putative CRPs, and more than 95% of the putative CRPs contain 12 or less cysteine residues (**Figure 1B**). The number of confirmed CRPs in the MtSSPdb were less than 50% of all putative CRPs, suggesting that potentially a large number of “unknown” SSPs need to be identified and characterized in wheat (**Figure 1C**).

**Figure 2:**
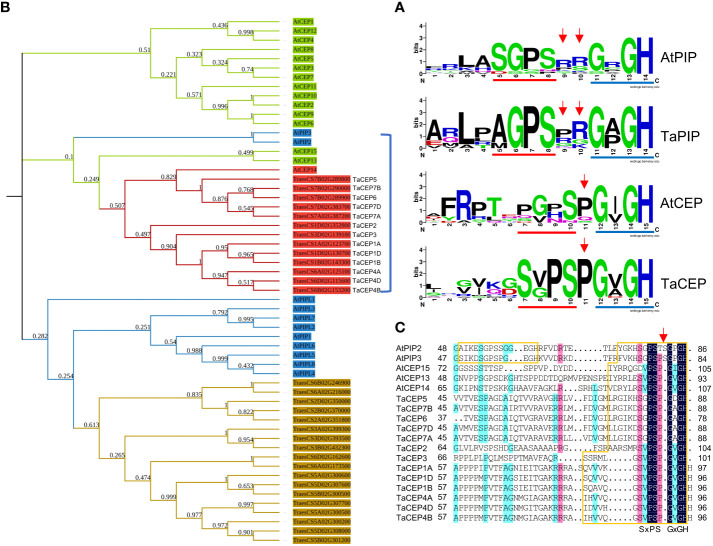
Identification of CEP family in wheat. **(A)** Logos of PIP and CEP peptides from Arabidopsis and wheat. Red and blue lines indicate SGPS and GxGH motif, red arrows indicate the amino acid insertions between these two domains. Logos were created by WebLogo online tool (https://weblogo.berkeley.edu/logo.cgi). **(B)** Phylogenetic tree of AtPIPs, AtCEPs, TaPIPs and TaCEPs, generated from the alignment of the full length protein sequences using ClustalW. The phylogenetic tree was constructed by the maximum likelihood method with 1000 bootstrap replications. Different colors indicate different peptide families in Arabidopsis and wheat. **(C)** Multiple sequence alignment of peptides within blue bracket from **(B)**. The SSP domains were boxed in orange, red arrow indicates the additional amino acid insertion in AtPIP peptides.

In exploring the unclassified TaSSPs, a conserved domain GVGH was identified at the C-termini of 13 putative TaSSPs. This domain is similar to part of the CEP and PIP domains, both of which contain two conserved domains SGPS and GxGH (x represents any amino acid residue) at the C-termini. For PIP peptides, two random amino acid residues are present between these two domains while for CEP peptides, a proline is in the middle (**Figure 2A**). To characterize this peptide family, together with CEP and PIP family members from *Arabidopsis*, C-terminal sequences of all the TaSSPs containing the GxGH domain were used in constructing a phylogenetic tree. The results showed that TaPIP and AtPIP members were clustered together in one branch, confirming the reliability of the prediction results from MtSSPdb. The unclassified TsSSPs were clustered together with AtCEP members (**Figure 2B**). The multiple sequence alignment of the branch containing the 13 GVGH-containing TaSSPs showed that the C-terminal sequences of the 13 TaSSPs were more conserved with AtCEPs, rather than AtPIPs (**Figure 2C**), suggesting that the 13 TaSSPs are members of the CEP peptide family.

**Figure 3:**
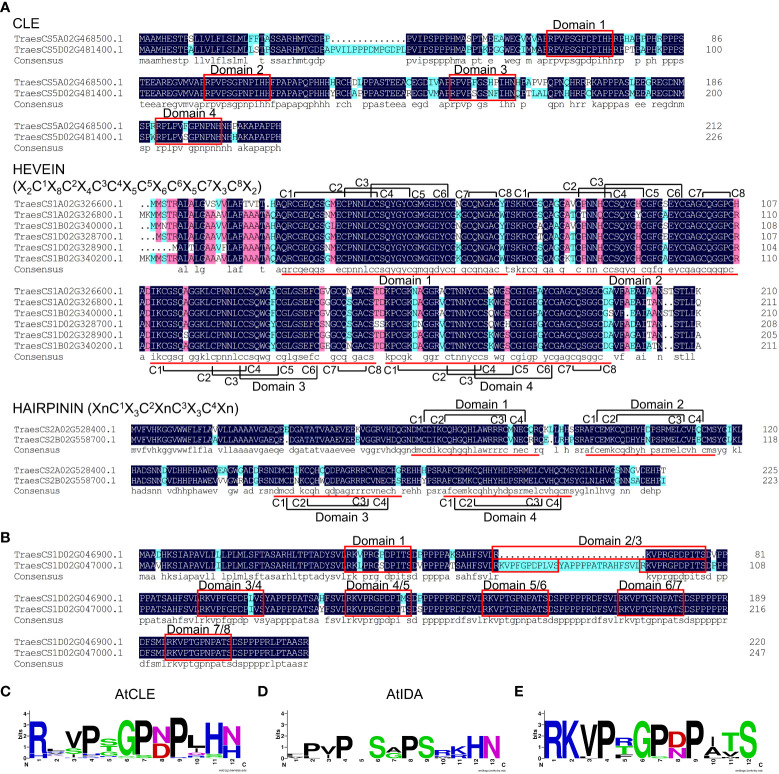
SSPs carrying multiple peptide domains in wheat. **(A)** Multiple sequence alignment of TaCLE, TaHEVEIN and TaHAIRPININ peptides carrying multiple SSP domains. Red boxes and lines indicate conserved SSP domains, black lines indicate the cysteines forming disulfide bonds, the sequences within brackets indicate the sequence models of HEVEIN and HAIRPININ peptides, respectively. **(B)** Multiple sequence alignment of TraesCS1D02G046900.1 and TraesCS1D02G047000.1 protein. Red boxes indicate conserved SSP domains. Multiple sequence alignment was performed using DNAMAN v6.0 software. **(C-E)** Logos of AtCLE motifs **(C)**, AtIDA motifs **(D)** and motifs in **(B)** and **(E)**, respectively. Logos were created by WebLogo online tool (https://weblogo.berkeley.edu/logo.cgi).

The phenomenon of a single gene encoding for multiple peptide domains has been reported in a number of plant species (Delay et al., 2013; Hou et al., 2014; Goad et al., 2017; Tian et al., 2021). In this study, a number of *TaSSP* genes were identified to encode multiple peptide domains, including CLE, HEVEIN and HAIRPININ domains (**Figure 3**). These include CLE, HEVEIN and HAIRPININ family genes encoding four corresponding peptide domains from the same family (**Figure 3A**), and two genes encoding seven and eight domains from different SSP families (**Figure 3B**). The MtSSPdb prediction puts these two muti-domain TaSSPs in the IDA family and the CLE family, respectively (**Table S2**). The sequence logos showed that the peptide domains of these two TaSSPs are more similar to CLE domains, except for the last two amino acids (HN/H), which have been reported to be essential for CLE peptide activity (**Figures 3C, D**) (Ito et al., 2006; Kondo et al., 2006). Similar peptide domains have been reported in a number of other plant species, including *P. virgatum*, *P. abies* and *A. lyrate*, and have been put into the “Others” group of the CLE family (Zhang et al., 2020). Whether they function as CLE peptides remains to be elucidated.

**Figure 4:**
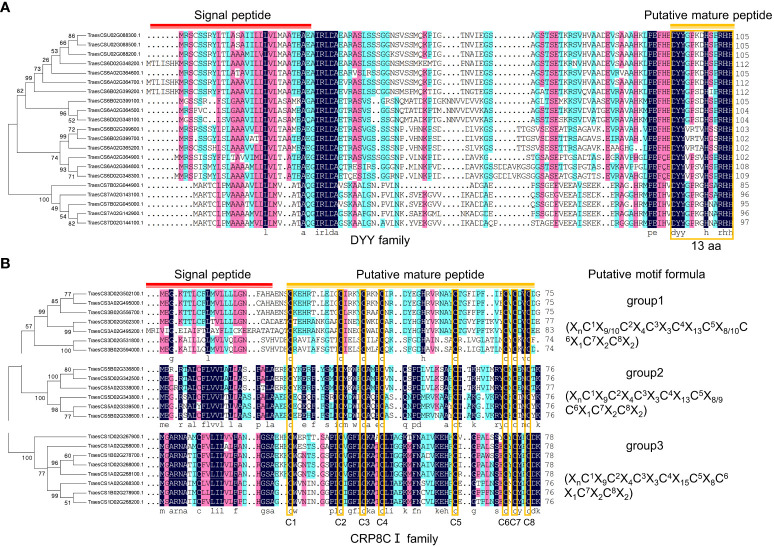
Potential novel SSP families in wheat. **(A)** DYY family consisting of 21 members. Phylogenetic tree was constructed using MEGA 5.0 software, multiple sequences alignment was performed using DNAMAN software. Red line indicates signal peptide, and orange line and box indicate putative mature peptide sequences consisting of 13 amino acids. **(B)** CRP8CI family, a CRP family consisting of 21 members. Phylogenetic tree was constructed using MEGA 5.0 software, multiple sequences alignment was performed using DNAMAN software. Red line indicates signal peptide, and orange line indicates putative mature peptide sequences containing eight cysteines, which are indicated with orange boxes. The sequences within brackets indicate motif formulas of each group.

In this study, two potentially novel TaSSP families were identified manually, named DYY family and CRP8CI family (**Figure 4**; **Table S3**). The DYY family is likely a PTM peptide family, with the first two amino acids of the conserved domain being ‘DY’, which is similar to the tyrosine sulfonated SSP families CIF, RGF/GLV/CLEL, PSK and PSY (**Figure 4A**). The CRP8CI family is a CRP family with 8 conserved cysteines in the putative SSP domain. The CRP8CI family is further divided into 3 groups basing on phylogenetic analysis and multiple sequences alignment (**Figure 4B**). No similar proteins of these two SSP families were found in Arabidospis. Further study is needed to elucidate the functions of the proteins in these two families as SSPs.

### Gene ontology analysis of SSPs in *T*. *aestivum*

**Figure 5:**
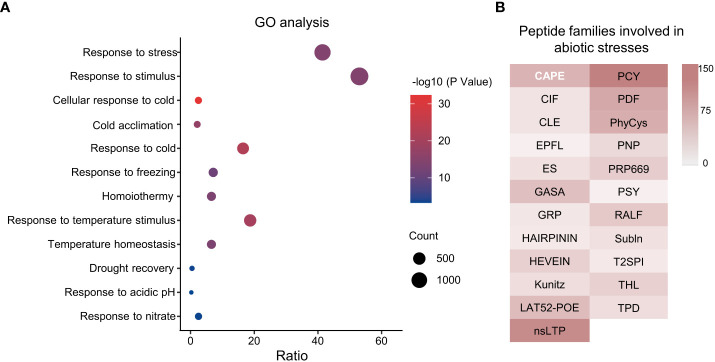
GO analysis of wheat SSPs. **(A)** Go terms involved in stress response. X-axis represents Gene Ratio of genes in each GO Terme to total gene numbers, Y-axis is GO Terms. The size of dots indicates the number of genes, and the color indicates P value. **(B)** Peptide families involved in abiotic stress response based on GO analysis. The color gradient indicates the number of genes.

To predict biological functions of TaSSPs, singular enrichment analysis (SEA) was performed for GO analysis using the agriGO online analysis tool (http://systemsbiology.cau.edu.cn/agriGOv2/index.php). Of the 4,981 putative TaSSPs, 2,665 had GO annotations, and 240 of the 1,365 GO terms were significant (with *P* value ≤ 0.001 and FDR ≤ 0.05), including abiotic stress responses such as response to stress and stimulus, cellular response to cold, response to temperature stimulus and drought recovery etc. (**Figure 5A**, **Table S4**). The GO annotations showed that 23 SSP families are possibly involved in abiotic stresses, including CAPE, CIF, CLE, HEVEIN, nsLTP, PDF, PSY and RALF etc. (**Figure 5B**). The results indicated potential functions of TaSSPs in responding to abiotic stresses.

### Expression patterns of *SSPs* in *T*. *aestivum*

**Figure 6:**
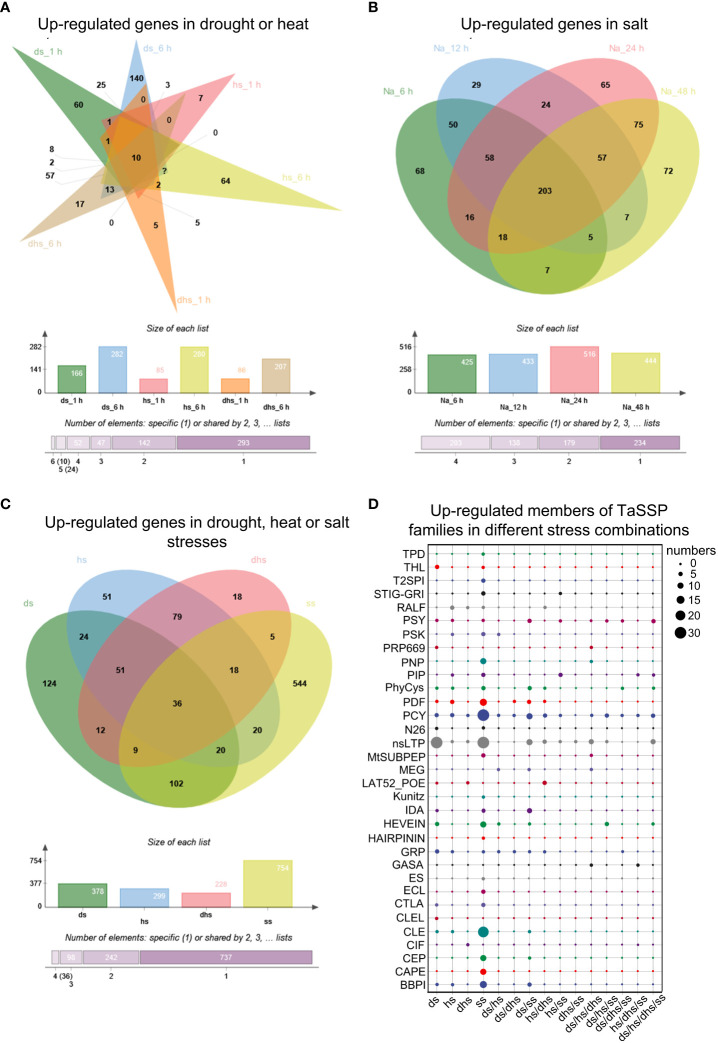
Expression analysis of *TaSSPs* under heat, drought and salt stress. **(A)** Upregulated *TaSSPs* in drought stress 1 h (ds_1 h), ds_6 h, heat stress1 h (hs_1 h), hs_6 h, drought and heat stress 1 h (dhs_1 h) and dhs_6 (h) **(B)** Upregulated *TaSSPs* in salt stress (ss) 6 h, 12 h, 24 h and 48 (h) Na indicates NaCl. **(C)** Upregulated *TaSSPs* in ds, hs or ss. **(D)** Upregulated members of TaSSP families in different stress combinations. The size of dots indicates the number of genes. The expression data was downloaded from WheatOmics 1.0 (https://wheatomics.sdau.edu.cn/).

To test our hypothesis that TaSSPs function in response to abiotic stresses, the RNA-seq data of wheat responding to drought, heat and salt stresses obtained from WheatOmics 1.0 was analyzed. Under drought, heat or drought plus heat stresses (ds, hs or dhs), 568 TaSSP-encoding genes are significantly upregulated under at least one of the above mentioned stresses (the TPM ratio of stress treatments to corresponding control ≥ 2), and among them, 166, 282, 85, 280, 86 and 207 *TaSSPs* are upregulated at ds_1 h, ds_6 h, hs_1 h, hs_6 h, dhs_1 h and dhs_6 h respectively. 10 *TaSSPs* are upregulated by all these stresses (**Figure 6A**; **Table S5**). Under salt stress (ss), 754 *TaSSPs* are significantly upregulated in total, and 203 *TaSSPs* are upregulated at all treating times (**Figure 6B**; **Table S6**).

Comprehensive analysis of the RNA-seq results from ds, hs and ss treatments revealed that 1113 *TaSSP* genes from 33 of the 38 TaSSP families are upregulated in drought, heat or salt stress (**Figures 6C, D**; **Table S7**), indicating the putative function of TaSSPs in response to abiotic stresses. Considering that *TaSSPs* might be upregulated under multiple stress conditions, the expression data was deduplicated and the results showed that 124, 51, 18 and 544 *TaSSPs* are specifically upregulated under ds, hs, dhs, or ss respectively, covering 31 of the 38 TaSSP families, such as the BBPI, CAPE, CEP, CLE, nsLTP, PCY, PIP and PSY families. 376 *TaSSPs* are upregulated by multiple stresses, covering 21 of the 38 SSP families, such as the CEP, GASA, GRP, HEVEIN, nsLTP, PCY, PDF and PIP families (**Figures 6C, D**; **Table S7**). These results indicates that most of the known TaSSP families might play a role in response to abiotic stresses, with many members responding to multiple stresses. Among the stress-responsive TaSSP families, 6 families have more than half members being upregulated under all stresses, including the CEP, IDA, N26, PIP, PSY and T2SPI families (**Figure S2**).

**Figure 7:**
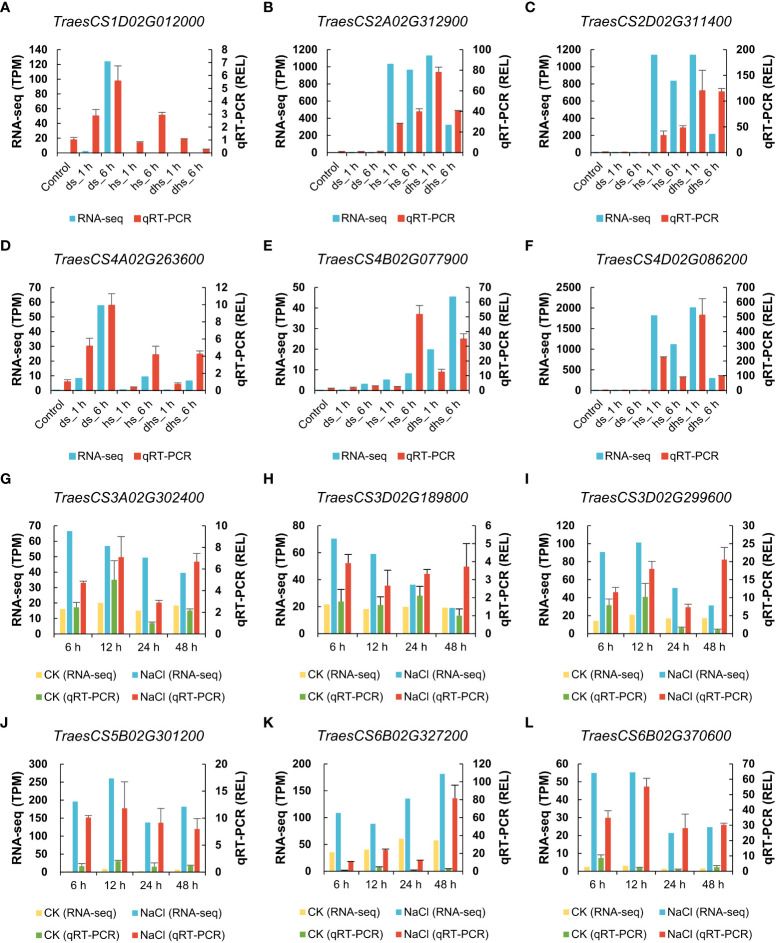
Verification of expression data from RNA-seq using qRT-PCR. **(A-F)** The expression level of *TaSSPs* in drought and heat stress. **(G-L)** The expression level of *TaSSPs* in salt stress. The highly expressed *TaSSP* genes were selected randomly for qRT-PCR. The left Y-axis indicates TPM value (Transcripts Per Kilobase Million) from RNA-seq in **(A-F)**, and TPM relative to control in **(G-L)**. The right Y-axis indicates relative expression level (REL) based on qRT-PCR. All samples used to perform qRT-PCR were collected based on corresponding samples for RNA-seq. qRT-PCR were performed three biological replicates and two technological replicates.

To verify the reliability of the above RNA-seq data, 12 *TaSSPs* were randomly selected for qRT-PCR analysis. The results showed that the expression patterns of these *TaSSPs* from qRT-PCR are consistent with the results of RNA-seq analysis (**Figure 7**).

### Exogenous application of TaCEP1D peptide enhanced drought tolerance of wheat plants

A previous report suggested that the CEP peptides mediated signaling was involved in abiotic stress tolerance in *Arabidopsis* (Smith et al., 2020; Taleski et al., 2022). In this study, RNA-seq data showed that 92.31% of the TaCEP family members are upregulated under all the tested stresses (**Figure S2**). For *in vitro* treatments, a peptide derived from the *TaCEP1D* gene *TraesCS1D02G130700* that was highly induced by drought and salt stresses was synthesized (**Figure S3**, **Table S1**). A preliminary detached leaf treatment experiment suggested that both 0.5 µM and 1.0 µM TaCEP1D peptide significantly alleviated the damage to detached leaves caused by drought or salt stress (**Figure S4**). Therefore 0.5 µM TaCEP1D peptide was used in the following peptide treatments. Previous studies indicated that CEP peptide treatments caused arrest of root growth and inhibited lateral root formation (Ohyama et al., 2008; Imin et al., 2013). In order to avoid the effects of CEP peptides on the root system to cause growth defects, TaCEP1D peptide was sprayed on the leaf surface of wheat plants.

**Figure 8:**
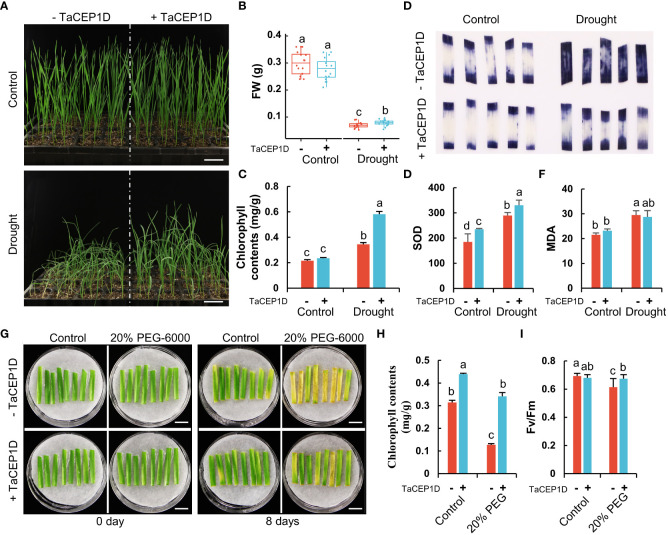
Exogenous application of TaCEP1D peptide altered drought response in wheat. **(A)** 2-week-old wheat plants treated with 0.5 µM TaCEP1D peptide for 4 days under control and drought condition. **(B-C)** Measurements of fresh weight (FW) and chlorophyll contents of wheat plants from **(A)**, respectively. **(D-F)** Nitroblue tetrazolium (NBT) staining of O^2-^ **(D)**, superoxide dismutase (SOD) activity detection **(E)**, measurement of molondialdehyde (MDA) contents **(F)**, respectively. Samples were harvested from the first leaves of plants from **(A)**. **(G)** Detached leaves treated with 0.5 µM TaCEP1D peptide for 8 days under 20% PEG-6000 simulated drought stress. The first leaves were cut from 2-week-old wheat plants cultured in Hoagland nutrient solution. **(H-I)** Measurements of chlorophyll contents and Fv/Fm of leaves from **(G)**, respectively. Sample size n = 16 in **(B)**, 3 in **(C, E, F, H)**, and 8 in **(I)**. All statistics analyses were performed three biological replicates. Different lowercase letters in **(B, C, E, F, H, I)** indicate statistically significant differences based on Student’s t-*test* (*p* < 0.05). Bar = 5 cm in **(A)**, and 1 cm in **(G)**.

In the drought stress treatments, tray-grown plants were subjected to water withdrawal. Four days after treatments, compared with the control plants which were sprayed with water, the plants treated with TaCEP1D peptide exhibited better performance, including firmer stalks, higher fresh weight and chlorophyll contents (**Figures 8A-C**). Without drought treatments, TaCEP1D peptide treatments did not cause detectable difference on the wheat plants (**Figures 8A-C**). These results suggest that TaCEP1D peptide treatments enhanced drought tolerance in wheat plants.

Stressed plants often have increased accumulation of reactive oxygen species (ROS) such as superoxide anion (O^2-^), which causes lipid peroxidation and cell damage. The results of nitroblue tetrazolium (NBT) staining showed that exogenous application of TaCEP1D peptide significantly reduced the accumulation of O^2-^ in leaves of wheat plants under drought stress, while no significant difference in ROS accumulation was observed in peptide-treated plants grown under normal growth conditions (**Figure 8D**). Furthermore, the activities of superoxide dismutase (SOD), an enzymatic antioxidant scavenging ROS, increased significantly after TaCEP1D peptide treatments, under both drought stress and control conditions (**Figure 8E**). Malondialdehyde (MDA), a metabolite of lipid peroxidation, showed increased accumulation in wheat plants under drought stress, but exogenous application of TaCEP1D peptide decreased the MDA level (**Figure 8F**). The results suggest that TaCEP1D peptide may function in reducing ROS accumulation in wheat plants under drought stress.

To further verify the role of TaCEP1D peptide in drought responses, detached wheat leaves were treated with PEG-6000 to simulate drought stress. The results showed that the detached leaves were sensitive to PEG-6000 treatments, showing significant reduction in chlorophyll content and maximum photochemical efficiency of PSII (Fv/Fm) 8 d after treatments. The PEG-6000 sensitivity of the detached leaves was significantly lower after exogenous application of TaCEP1D peptide (**Figures 8G-I**), suggesting that TaCEP1D peptide significantly increased the tolerance of detached leaves to PEG-6000.

### Exogenous application of TaCEP1D peptide enhanced the tolerance of wheat plants to salt stress

**Figure 9:**
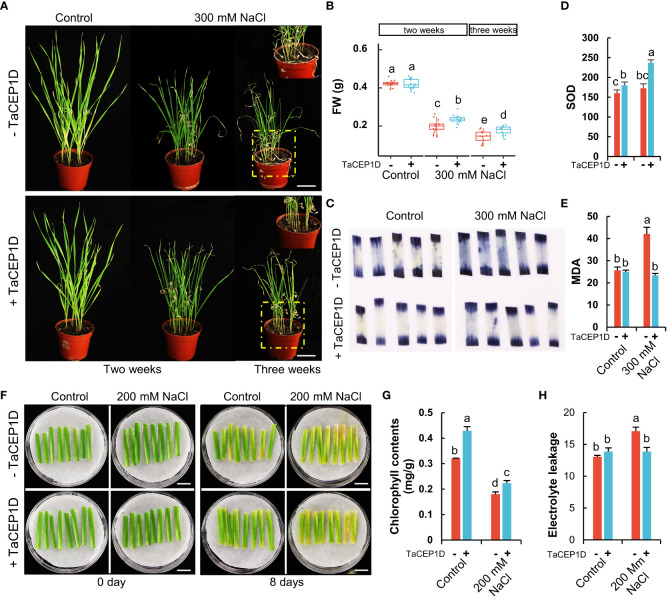
Exogenous application of TaCEP1D peptide altered salt stress response in wheat. **(A)** Wheat plants treated with 0.5 µM TaCEP1D peptide for 2 and 3 weeks under control and 300 mM NaCl conditions. **(B)** Measurements of fresh weight (FW) of wheat plants from **(A)**. **(C-E)** Nitroblue tetrazolium (NBT) staining of O^2-^ **(C)**, superoxide dismutase (SOD) activity detection **(D)**, measurement of molondialdehyde (MDA) contents **(E)**, respectively. Samples were harvested from the first leaves of plants from **(A)**. **(F)** Detached leaves treated with 0.5 µM TaCEP1D peptide for 8 days with 200 mM NaCl. The first leaves were cut from two-week-old wheat plants cultured in Hoagland nutrient solution. **(G-H)** Measurements of chlorophyll contents and electrolyte leakage of leaves from **(G)**, respectively. Sample size n = 16 in **(B)** and 3 in **(D, E, G, H)**. All statistics analyses were performed with three biological replicates. Lowercase letters in **(B, D, E, G, H)** indicate statistically significant difference based on Student’s t-*test* (*p* < 0.05). Bar = 5 cm in **(A)**, and 1 cm in **(F)**.

In addition to drought, the *TaCEP1D* gene is also significantly upregulated by salt stress (**Figure S3**). To study the role of TaCEP1D peptide in salt stress response, pot-grown wheat plants were treated using 300 mM NaCl. The growth of wheat plants was seriously inhibited two weeks after salt application, and more significant growth inhibition was observed three weeks after treatments (**Figure 9A**). The plants treated with TaCEP1D peptide exhibited tolerance to salt stress compared with the untreated plants, with significantly healthier leaves and higher fresh weight (**Figures 9A, B**). NBT staining showed that exogenous application of TaCEP1D peptide significantly reduced the accumulation of O^2-^ in leaves of wheat plants under salt stress, while no significant difference between treated and untreated plants was observed under normal growth conditions (**Figure 9C**). Furthermore, SOD activities were significantly higher in TaCEP1D peptide treated plants, under both salt stress and control conditions (**Figure 9D**). In salt-stressed wheat plants MDA contents were significantly increased, but exogenous application of TaCEP1D peptide reduced MDA content to the levels of unstressed plants (**Figure 9E**). The results suggest that TaCEP1D peptide might enhance the tolerance of wheat plants to salt stress through reducing ROS accumulation.

When wheat detached leaves were treated by 200 mM NaCl with or without TaCEP1D peptide, peptide treatments alleviated the damage from salt stress (**Figure 9F**) with higher chlorophyll contents (**Figure 9G**) and less electrolyte leakage (**Figure 9H**). The results further suggested that TaCEP1D functions in enhancing salt tolerance of wheat.

## Discussion

### Identification of SSPs in plants

Totally 4,981 putative SSPs were identified in wheat, among which 1,704 SSPs were predicted in MtSSPdb and 86 more SSPs were predicted by manual verification (**Table S2**). In this study, the current database publicly available for SSPs prediction *in silico*, MtSSPdb, failed in predicting some of the peptide families including the TaCEP family (**Figure 2**). The BLAST method is often used to predict members of the known SSP families. For example, the poplar CLEL family and the switchgrass CLE family were identified *via* BLAST (Tian et al., 2019; Tian et al., 2021). Another common approach for SSPs prediction is HMMER (Eddy, 1998). For both BLAST and HMMER, appropriate thresholds need to be defined, such as *S* score and *E*-*value* for BLAST, and the training set for HMMER. Although there have been attempts to develop new methods for SSP prediction (Zhang et al., 2020), a comprehensive and accurate database for SSPs prediction is needed for SSP prediction.

In this study, 64% (3,191 of 4,981) of the putative TaSSPs were not confirmed as peptides based on currently known peptide domains (**Figure 1A**). A large number of plant genes likely encoding SSPs need to be excavated and identified. For example, two novel SSP families, DYY and CRP8CI peptides, have been identified in this study (**Figure 2**). Furthermore, based on cysteine residue analysis, TaCRPs accounted for 70% of total putative TaSSPs (**Figure 1A**), but 55% CRPs were not predicted as known peptides (**Figure 1C**). A large number of putative TaCRPs were shown to be upregulated under stresses (**Figure 6D**, **Tables S5**-**7**). We found that chemical synthesis of CRPs is difficult due to the disulfide bonds, which are easily oxidized. As a result, the quick approach of exogenous peptide application often used in studying peptide functions can not be readily used for CRPs, posing even bigger challenges in identification and characterization of these SSPs.

### SSPs containing multiple domains

Goad et al. reported 59 genes encoding multiple CLE domains from 27 plant species (Goad et al., 2017), indicating that CLE containing multiple domains are common in plants. Some members of Arabidopsis CEP and PIP families also contain multiple peptide domains (Delay et al., 2013; Hou et al., 2014). Fewer multiple domain-containing CRPs (Cysteine rich peptides) were identified, including the HEVEIN-LIKE gene in *Stellaria. Media* L. which encodes two HEVEIN-LIKE domains (Slavokhotova et al., 2017). In this study, we have identified a number of TaSSPs containing multiple peptide domains, including CLE domains, HEVEIN domains, HAIRPININ domains and several unknown peptide domains (**Figure 3A**). No *TaCEP* or *TaPIP* genes encoding multiple domains were identified in this study. The identification of genes encoding multiple HEVEIN and HAIRPININ domains suggests that multiple-domain CRPs may be also common in plants. Due to number and positional uncertainty of peptide domains, and inconsistency between peptide domain sequences, currently SSPs containing multiple peptide domains can only be identified manually, Interestingly, we found some variation in multiple-domain SSPs among plant species. For example, SSPs with multiple CLE domains exist widely in gramineous plants, such as rice, wheat and switchgrass. Single peptide domains are usually localized at the C-terminus of the proproteins, while multiple peptide domains often occurred in the variable domain of SSPs. Search for peptide domains in the variable domain of putative SSPs may help in identifying SSPs contains multiple peptide domains.

Limited studies on the function of SSP proteins carrying multiple peptide domains have been reported. Oelkers et al. hypothesized that multiple-domain CLE protein precursors can release several active peptides after processing, and may play an amplification effect (Oelkers et al., 2008). Our previous study showed that the TDIFL activity was not enhanced by heterologous expression of *P. virgatum TDIFL* genes in *Arabidopsis*, which encode multiple TDIFL domains (Tian et al., 2021). It will be interesting to find out how different peptides released from the same multi-domain *SSP* gene coordinate in exerting their functions.

### The potential functions of TaSSPs in stress responses

The results from GO analysis and expression patterns under abiotic stresses of TaSSPs (**Figure 5**, **6**) suggest that a large number of TaSSPs might be involved in stress response. Several peptides have been reported to play a role in response to abiotic stresses, including RALFL8 responding to drought stress (Atkinson et al., 2013), CLE25 responding to dehydration stress (Takahashi et al., 2018), CAPE1 and PIP3 responding to salinity stress (Chien et al., 2015; Zhou et al., 2022), and CEP5 responding to osmotic and drought stresses (Smith et al., 2020). A large number of *TaSSP*s from RALFL, CLE, CEP, PIP, and CAPE families showed upregulated expression under abiotic stresses (**Figure 6D**), indicating potential functions of TaSSPs in wheat’s response to abiotic stresses.

Previous studies have demonstrated that CEP peptide family members are involved in primary and lateral root growth and development, enhancement of nodulation, aboveground Arabidopsis growth and development (Ohyama et al., 2008; Imin et al., 2013; Roberts et al., 2013; Mohd-Radzman et al., 2015; Taleski et al., 2018). Recently, CEP5 was reported to be involved in osmotic and drought stress tolerance in *Arabidopsis* (Smith et al., 2020). In this study, we showed that exogenous application of the TaCEP1D peptide encoded by *TraesCS1D02G130700* enhanced the tolerance of wheat plants and detached leaves to drought and salt stresses (**Figures 8**, **9**). However, a significant difference of NBT staining was not observed in Control+TaCEP1D compared with NaCl+TaCEP1D leaves (**Figure 9C**), we suspect that the growth and development of wheat seedlings under stress were severely inhibited compared to control plants, resulting in a more mature development stage of leaves of control plants. Application of biosynthesized peptides in agriculture has been reported (Zhang and Gleason, 2020). Unlike phytohormones which are generally involved in multiple developmental and stress response processes, SSPs appear to be more specific in their functions. In this study, we found that TaCEP1D peptide had no significant effects on plant height or other traits other than stress tolerance (**Figures 8**, **9**). Peptide-based growth regulators have great potential in agricultural application.

SSP signals need to be perceived by corresponding receptors to initiate signal transduction. XYLEM INTERMIXED WITH PHLOEM 1 (XIP1)/CEP RECEPTOR 1 (CEPR1) and CEPR2 have been proposed to be the receptors for CEP peptides during lateral root initiation (Tabata et al., 2014). However, whether CEP peptides’ function in response to drought and salt stresses is dependent on CEPR1/CEPTR2 remains unknown. The PIP3-RLK7 signal module has recently been reported to regulate plant salt tolerance in *Arabidopsis* (Zhou et al., 2022). Intriguingly, CEPR1, CEPR2 and RLK7 are highly similar in protein sequences and belong to the same subgroup of the LRR-RLK subclass XI. CEPs and PIPs also share high similarity in domain sequences (**Figure 2**), suggesting that CEP peptides regulating wheat drought and salt stress tolerance might also depend on RLK7.

## Data availability statement

The datasets presented in this study can be found in online repositories. The names of the repository/repositories and accession number(s) can be found in the article/**Supplementary Material**.

## Author contributions

YG, IS and TL conceived the research. DT and YG designed the experiments. DT, QX, BZ, and YD performed the bioinformatic analyses. DT, ZD, JX, WL and ZZ performed the experiments. DT and YG analyzed the data. DT drafted the manuscript. YG, IDS and TL revised the manuscript. All authors contributed to the article and approved the submitted version.

## Funding

This work was supported by funding from National Natural Science Foundation of China (Nos. 31970204), and the Postdoctoral Applied Research Project of Qingdao, China, and the Agricultural Science and Technology Innovation Program, Chinese Academy of Agricultural Sciences (ASTIP-TRI02), China.

## Conflict of interest

The authors declare that the research was conducted in the absence of any commercial or financial relationships that could be construed as a potential conflict of interest.

## Publisher’s note

All claims expressed in this article are solely those of the authors and do not necessarily represent those of their affiliated organizations, or those of the publisher, the editors and the reviewers. Any product that may be evaluated in this article, or claim that may be made by its manufacturer, is not guaranteed or endorsed by the publisher.

## Supplementary material

Supplementary Figure 1 Identification procedure and chromosomes distribution of SSPs in wheat. **(A)** The process of SSPs identification in wheat. The numbers in brackets indicate the numbers of identified SSPs in each step. The diagram on the right indicates the screening conditions in each step. ‘X’ indicates deletion. The wheat sequences were downloaded from *EnsemblPlants* (https://plants.ensembl.org/info/data/ftp/index.html). **(B)** The number of SSPs with different protein length ranges. **(C)** Chromosomes distribution of SSPs. The up graph shows the location of each SSP in different chromosomes, and the lower graph shows the percentage of SSPs to total proteins located on each chromosome.

Supplementary Figure 2 The proportion of stress upregulated genes in each TaSSP family.* TaSSP families with more than 50% members being upregulated in stresses.

Supplementary Figure 3 Expression pattern of TaCEP family genes. (**A**, **B**) Heatmap of *TaCEPs* based on the RNA-seq data under drought and heat stress, salt stress, respectively. (**C**, **D**) Expression level of *TaCEP1A, 1B* and *1D*.

Supplementary Figure 4 TaCEP1D peptide treatments of detached leaves under drought and salt stresses. (**A**) Detached wheat leaves before treatments. (**B**) Detached wheat leaves treated with 0.5 µM and 1.0 µM TaCEP1D peptide under drought and salt stress for 6 days, respectively. 20% PEG-6000 was used to simulate drought stress. (**C**) Chlorophyll contents. Three biological replicates were performed for each treatment. Sample size n = 10. Lowercase letters indicate significant differences with *p* < 0.05. Bar = 1 cm in (**A**, **B**).
