# Corrigendum: Small secreted peptides encoded on the wheat (*Triticum aestivum* L.) genome and their potential roles in stress responses

**DOI:** 10.3389/fpls.2022.1064486

**Published:** 2022-11-18

**Authors:** Dongdong Tian, Qi Xie, Zhichao Deng, Jin Xue, Wei Li, Zenglin Zhang, Yifei Dai, Bo Zheng, Tiegang Lu, Ive De Smet, Yongfeng Guo

**Affiliations:** ^1^ Tobacco Research Institute, Chinese Academy of Agricultural Sciences, Qingdao, China; ^2^ Key Laboratory of Horticultural Plant Biology of Ministry of Education, College of Horticulture and Forestry Science, Huazhong Agricultural University, Wuhan, China; ^3^ Biotechnology Research Institute, Chinese Academy of Agricultural Sciences, Beijing, China; ^4^ Department of Plant Biotechnology and Bioinformatics, Ghent University, Ghent, Belgium; ^5^ VIB Center for Plant Systems Biology, Ghent, Belgium

**Keywords:** small secreted peptides, wheat, stress response, drought stress, salt stress, heat stress

In the published article, a second affiliation was omitted for author Ive De Smet. The correct affiliation details for Ive De Smet appear as follows:

4 Department of Plant Biotechnology and Bioinformatics, Ghent University, Ghent, Belgium
5 VIB Center for Plant Systems Biology, Ghent, Belgium

In the published article, there was an error in the article title: “Small secreted peptides encoded on the wheat (triticum aestivum L.) genome and their potential roles in stress responses”. The correct title appears as follows:

Small secreted peptides encoded on the wheat (Triticum aestivum L.) genome and their potential roles in stress responses

The authors apologize for these errors and state that this does not change the scientific conclusions of the article in any way. The original article has been updated.

## Publisher’s note

All claims expressed in this article are solely those of the authors and do not necessarily represent those of their affiliated organizations, or those of the publisher, the editors and the reviewers. Any product that may be evaluated in this article, or claim that may be made by its manufacturer, is not guaranteed or endorsed by the publisher.

