# Supplementary figures and images for: Small secreted peptides encoded on the wheat (Triticum aestivum L.) genome and their potential roles in stress responses
Source: Front Plant Sci. 2022 Sep 21;13:1000297. doi: 10.3389/fpls.2022.1000297 (PMC9532867; doi:10.3389/fpls.2022.1000297)

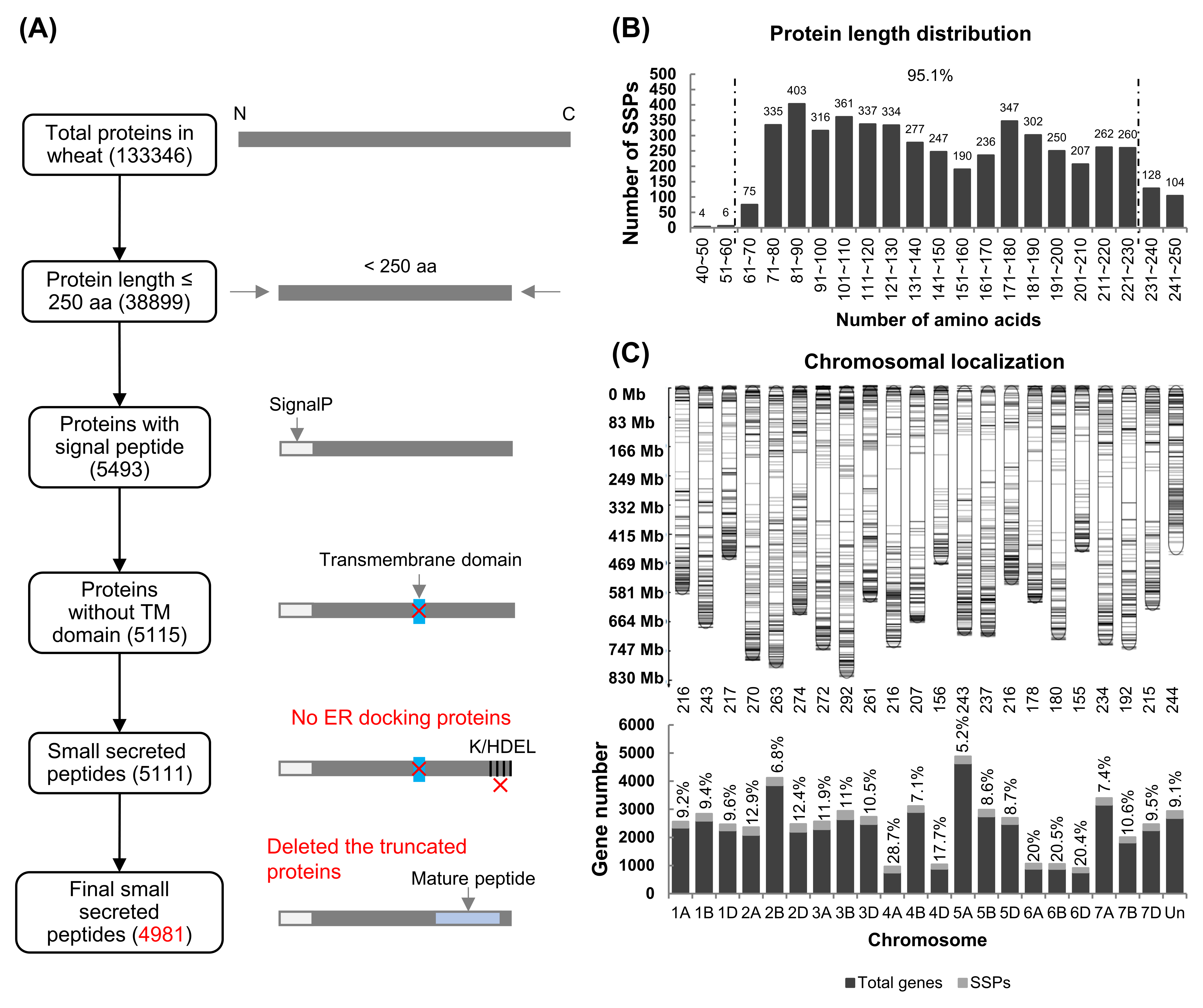

Supplement: Supplementary Figure 1 — Identification procedure and chromosomes distribution of SSPs in wheat. (A) The process of SSPs identification in wheat. The numbers in brackets indicate the numbers of identified SSPs in each step. The diagram on the right indicates the screening conditions in each step. ‘X’ indicates deletion. The wheat sequences were downloaded from EnsemblPlants (https://plants.ensembl.org/info/data/ftp/index.html). (B) The number of SSPs with different protein length ranges. (C) Chromosomes distribution of SSPs. The up graph shows the location of each SSP in different chromosomes, and the lower graph shows the percentage of SSPs to total proteins located on each chromosome. [file Image_1.tif]

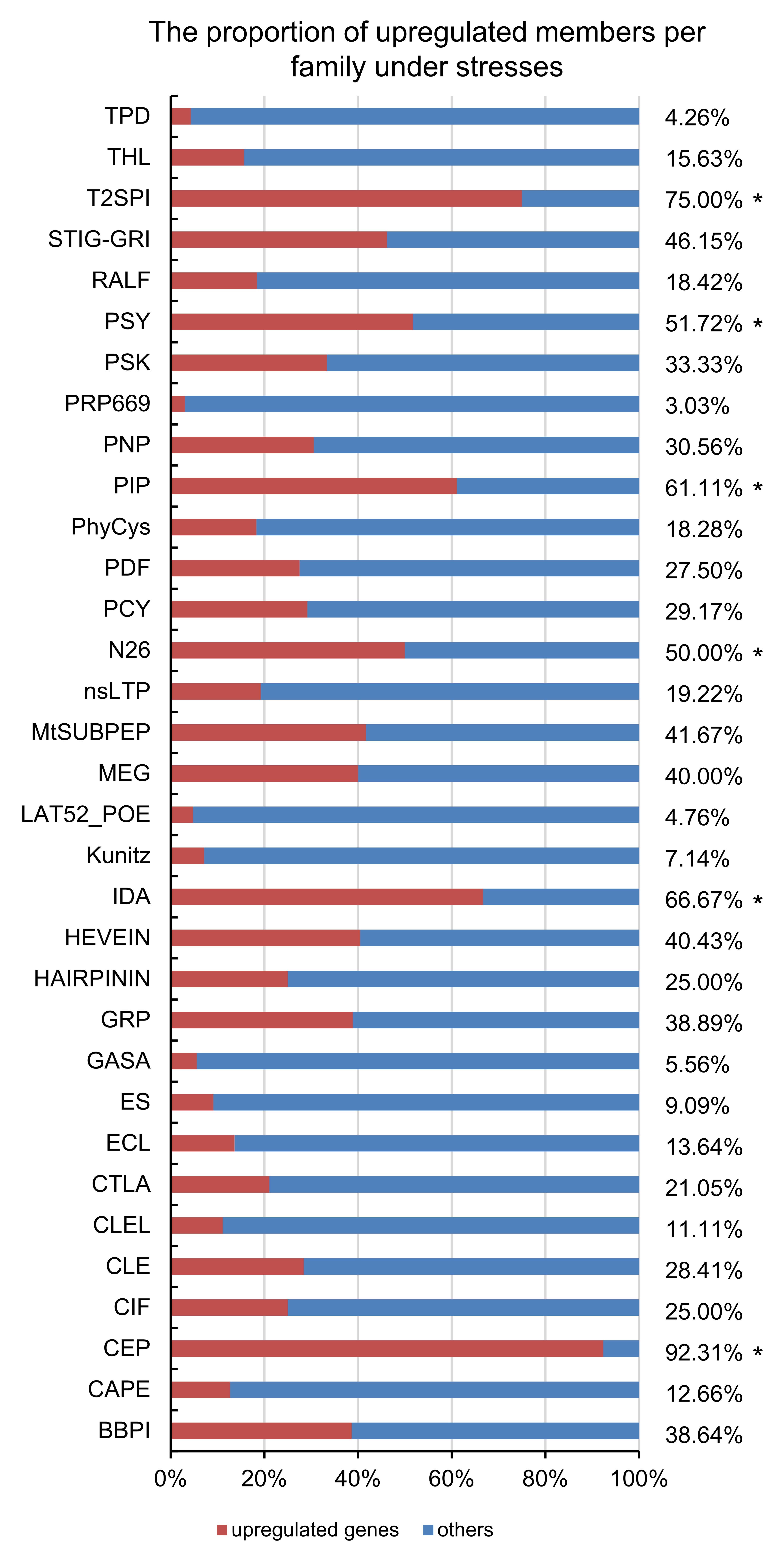

Supplement: Supplementary Figure 2 — The proportion of stress upregulated genes in each TaSSP family.* TaSSP families with more than 50% members being upregulated in stresses. [file Image_2.tif]

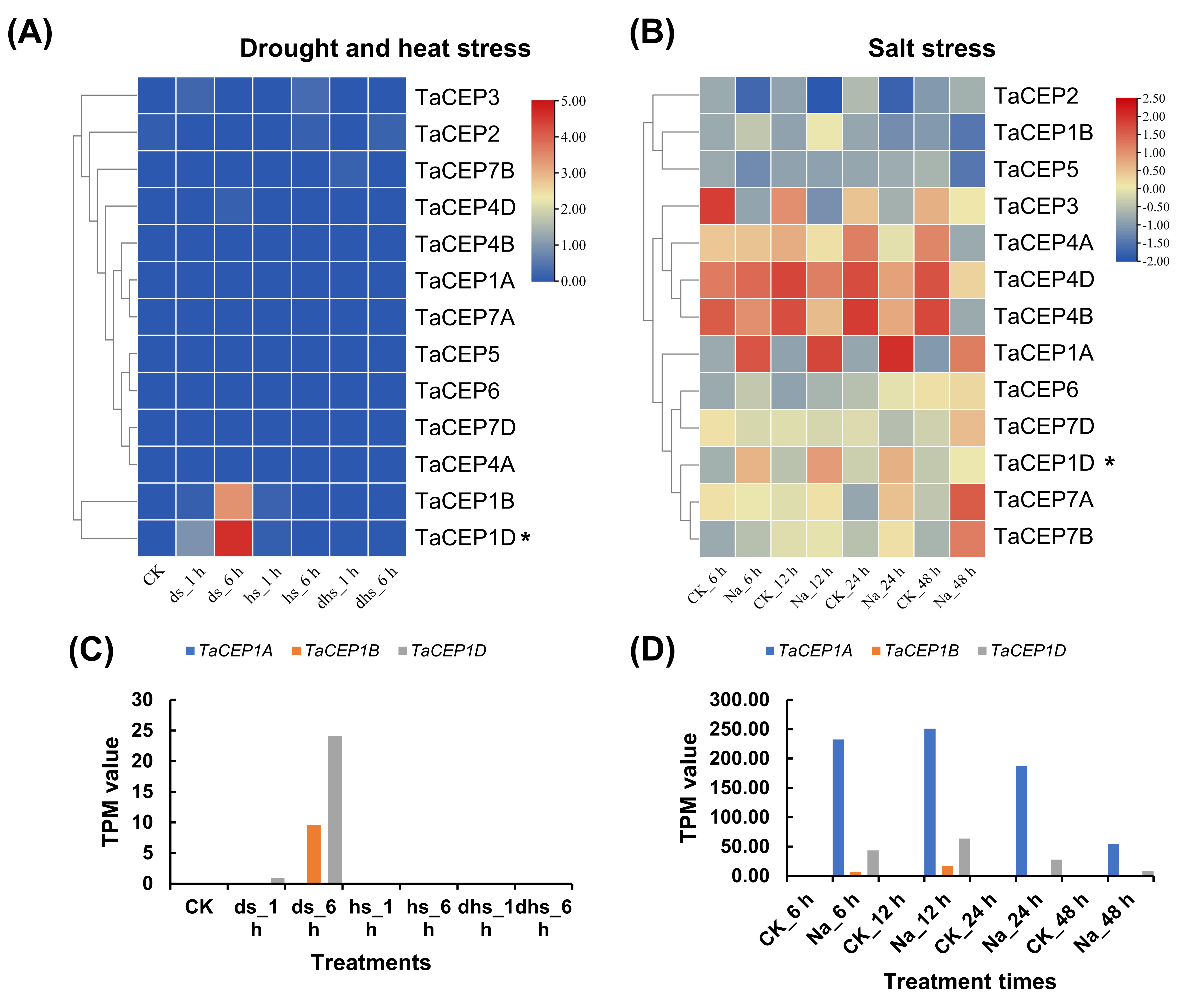

Supplement: Supplementary Figure 3 — Expression pattern of TaCEP family genes. (A, B) Heatmap of TaCEPs based on the RNA-seq data under drought and heat stress, salt stress, respectively. (C, D) Expression level of TaCEP1A, 1B and 1D. [file Image_3.tif]

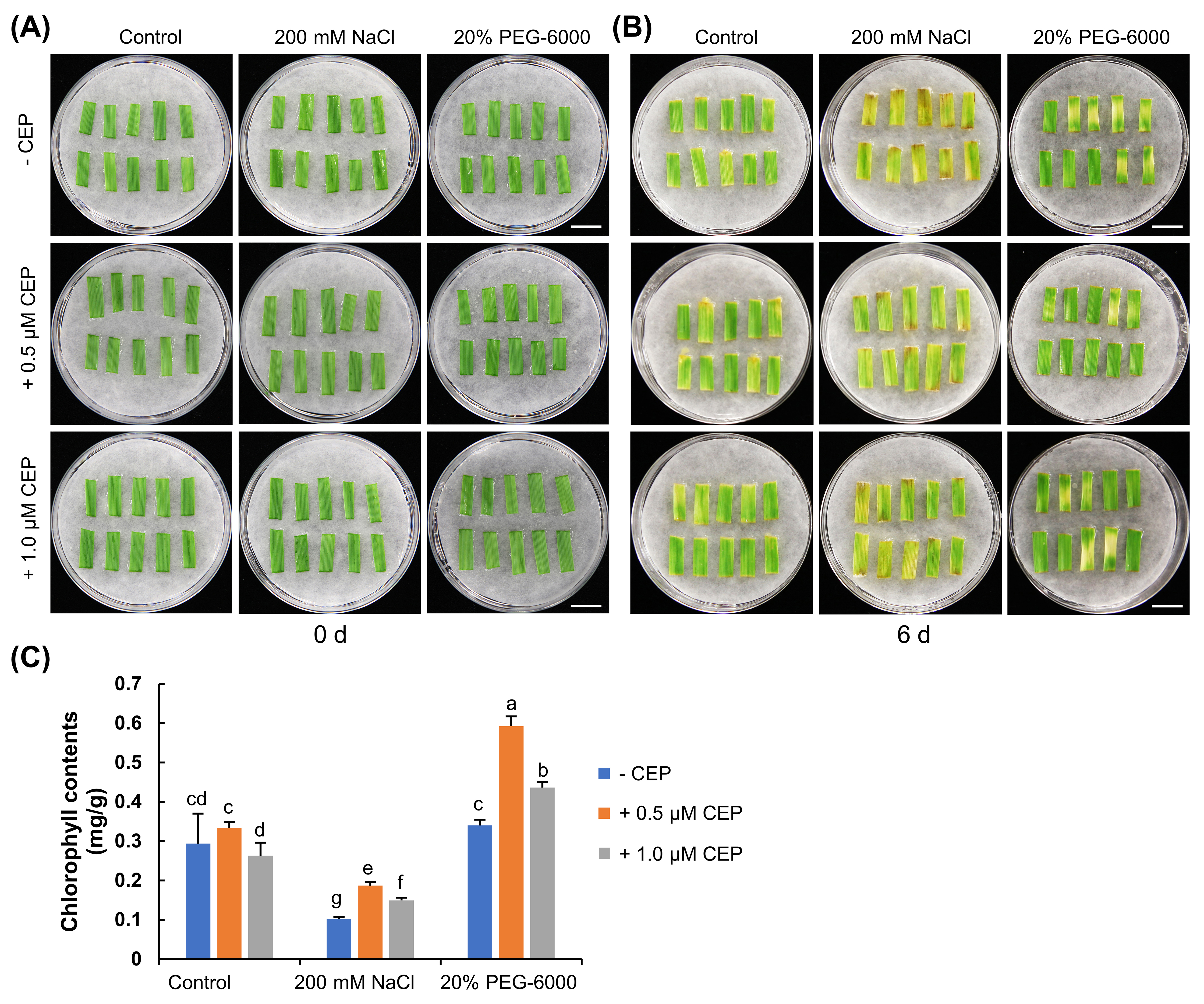

Supplement: Supplementary Figure 4 — TaCEP1D peptide treatments of detached leaves under drought and salt stresses. (A) Detached wheat leaves before treatments. (B) Detached wheat leaves treated with 0.5 µM and 1.0 µM TaCEP1D peptide under drought and salt stress for 6 days, respectively. 20% PEG-6000 was used to simulate drought stress. (C) Chlorophyll contents. Three biological replicates were performed for each treatment. Sample size n = 10. Lowercase letters indicate significant differences with p < 0.05. Bar = 1 cm in (A, B). [file Image_4.jpeg]
