# Supplementary material for: Small secreted peptides encoded on the wheat (Triticum aestivum L.) genome and their potential roles in stress responses
Source: Front Plant Sci. 2022 Sep 21;13:1000297. doi: 10.3389/fpls.2022.1000297 (PMC9532867; doi:10.3389/fpls.2022.1000297)
Supplement: Supplementary file 5 [file Table_1.docx]

| **Gene symbol** | **Forward primer** | **Reverse primer** | **Product length (bp)** |
| --- | --- | --- | --- |
| **qRT-PCR primers** |  |  |  |
| TraesCS1D02G012000 | AGTCGCAGAGCCACAAGTTCA | GTAGCACTTGCGCTCGACGTA | 108 |
| TraesCS2A02G312900 | GGATGAGGAAGGAGGACCTAAAG | CCCTTCCGTTCTTCCACCTC | 94 |
| TraesCS2D02G311400 | ATGCTTGTTGGCGCTTGG | TCAGGTCCTCCTTCCTCATCC | 308 |
| TraesCS4A02G263600 | AGCTCAAGATCAACGTGCCG | GCCGCAGTAGTTGAGGACGA | 164 |
| TraesCS4B02G077900 | GACGCTGCTTCAGGGTTCT | CGCAGTACTTCATGCACGC | 88 |
| TraesCS4D02G086200 | AGGTGCGACTGGAAGGAGACA | CTCCTCTACCTCCACCTTCACATC | 93 |
| TraesCS3A02G302400 | AGCGGAACAGCCAGACCAA | CACTTGCGGTTGCAGGGTAT | 108 |
| TraesCS3D02G189800 | GACGCCACCTCCTTCACCAT | TCACGCCATCCAGGCTCAT | 68 |
| TraesCS3D02G299600 | CCTACGCGGTCAACAGCTACTA | GTACTTGCAGGTTCCCGAGCT | 116 |
| TraesCS5B02G301200 | CGCTCGTGGTCGTCTTTCTG | AGCATCTCCACCGTCTCCCT | 154 |
| TraesCS6B02G327200 | TCCTTGTTGTCCTGGGTTGC | TTGTCTCCAACTGCGAAGGTC | 130 |
| TraesCS6B02G370600 | TTCTACCTCGTGCTGCAGTG | CAGTTCTGCGGGTAGGTTC | 143 |
| ACT2 (TC234027)^a^ | CAAATCATGTTTGAGACCTTCAATG | ACCAGAATCCAACACGATACCTG | 108 |
| CYP18-2 (AY456122.1)^a^ | GATCTCCGTGGTTGGTTTAGGA | CGCCGGACACAGATCCA | 123 |
| TaFNR1I (AJ457980.1)^a^ | CACCGGCCCAGTGATCTT | AAGGGCGTCTGCTCCAACT | 69 |
| ACT (AB181991)^a^ | GGAGAAGCTCGCTTACGTG | GGGCACCTGAACCTTTCTGA | 136 |
| UBI (AY297059)^a^ | GGAGTCCACCCTTCACTTGG | GACACAGGCACCATTCGAG | 130 |
| **Peptides synthesis** |  | **Mature peptide sequence** | |
| TaCEP1D |  | SQVVQGSVPSPGVGH | |

Table S1. Primers for qRT-PCR and synthetic peptide sequences in this study.

^a^ indicates the GenBank accession number of corresponding reference genes.
